# Mantle cell lymphoma-like solitary polypoid tumor of the esophagus: a case report

**DOI:** 10.1186/1757-1626-2-6646

**Published:** 2009-05-15

**Authors:** Takahiro Mori, Takuya Komeno, Haruo Ohtani

**Affiliations:** 1 Department of Surgery280 Sakuranosato, Ibaraki, 311-3193Japan; 2 Department of Hematology280 Sakuranosato, Ibaraki, 311-3193Japan; 3 Department of Pathology, National Hospital Organization Mito Medical Center280 Sakuranosato, Ibaraki, 311-3193Japan

## Abstract

**Introduction:**

Primary mantle cell lymphoma of the esophagus is quite rare, and we report here a case of a submucosal polypoid tumor of the esophagus that was pathologically similar, but not identical, to mantle cell lymphoma.

**Case presentation:**

A 66-year-old man underwent surgery in our hospital for a submucosal tumor of the esophagus. Histopathologically, the submucosal tumor uniformly showed a vague, nodular pattern composed of a regular proliferation of CD3-CD10-CD20+CD79a+bcl2+ small lymphoid cells with islands of abortive CD10+Ki67+ germinal centers, without evidence of marginal zone formation or lymphoepithelial lesions. These features were consistent with mantle cell lymphoma. However, the proliferating cells weakly expressed the D-type cyclins, IgD and IgM. The tumor was diagnosed as a “mantle cell lymphoma-like tumor”. Postoperative examination confirmed no tumour involvement in other organs. The patient was treated with rituximab postoperatively and has been disease free for more than 28 months after surgery.

**Conclusion:**

The present case might be regarded as a “hyperplastic change of the mantle zone” without the molecular features of mantle cell lymphoma, a case that adds to the limited clinicopathological data on B-lymphoproliferative diseases of the esophagus.

## Introduction

The esophagus is the least common site of involvement of lymphoma in the digestive tract, accounting for less than 1% of lymphoma patients. Primary esophageal lymphomas are usually large B-cell types or low-grade B-cell mucosa-associated lymphoid tissue (MALT) [1]. Furthermore, primary mantle cell lymphoma (MCL) of the esophagus has not been reported, only a case of MCL arising in Barrett's dysplasia [2]. Here we report an esophageal polypoid tumor that resembles MCL but lacks critical MCL features.

## Case presentation

**Figure 1.**
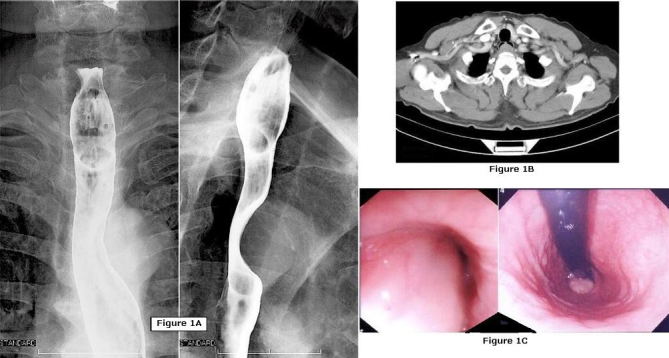
Preoperative examinations. An esophageal submucosal polypoid lesion was identified by barium esophagogram **(A)**, CT **(B)**, and downward (**C**, left) and upward (**C**, right) views by upper GI endoscope.

A 66 year-old Japanese male underwent upper gastrointestinal endoscopy by his practitioner for complaints of dysphagia, which revealed an esophageal submucosal tumor. He was referred to the National Hospital Organization Mito Medical Center in 2006 for treatment. He did not complain of fever, weight loss, or night sweating. On admission, physical examination revealed no remarkable features; the liver, spleen, and superficial nodes were not palpable. His laboratory data showed no abnormalities except a 10-year history of abnormal glucose tolerance treated by his primary physician. No elevated tumor markers or autoimmune antibodies were observed. A barium esophagogram, CT, and upper GI endoscope showed an esophageal submucosal polypoid lesion at the cervical esophagus (Figure 1). Its location close to the esophageal orifice and large size (4 × 2 cm) made an endoscopic resection technically difficult, and the patient was enrolled in the Department of Surgery. As the tumor was endoscopically diagnosed as benign leiomyoma or lipoma, we decided to perform a tumor resection because of the patient's complaints of dysphagia and because the cervical esophagus is relatively approachable compared with the thoracic esophagus, even without pathological diagnosis. The tumor was removed through a cervical esophagotomy under a cervical collar incision.

**Figure 2.**
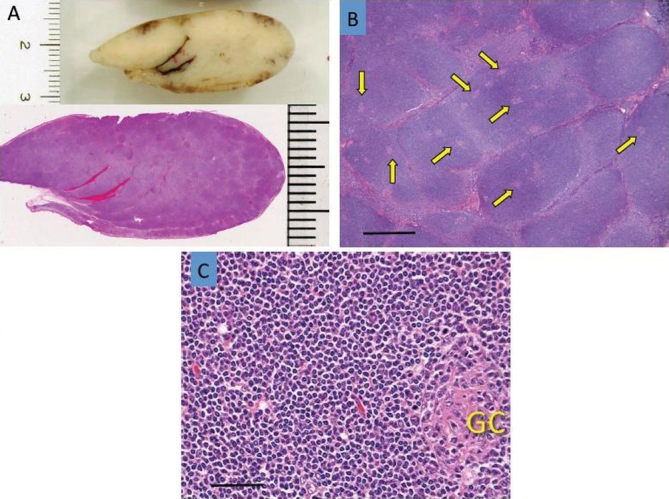
Resected specimen. Macroscopic appearance of the tumor **(A)**, low magnification of the polypoid lesion **(B)**, and microscopic image of atypical lymphoid cells **(C)**. Vague nodules with an abortive germinal center are indicated by arrows. Scale bar = 1 mm **(B)**. Regular small lymphoid cells surrounding abortive germinal centers. Scale bar = 50 μm **(C)**.

**Figure 3.**
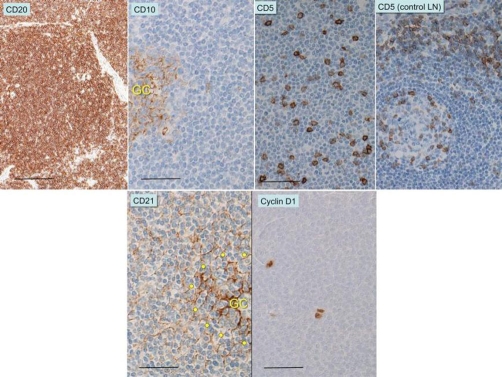
Immunohistochemical analyses of the tumor. CD20-positive lymphoid cells surround abortive CD10-positive germinal centers. Scale, 200 μm or 50 μm, is indicated by bars in CD20 or CD10, respectively. About 10-20% of proliferating cells were CD5 positive lymphoid cells (T-cells), but the vast majority were not. CD21-positive follicular dendritic cells were structurally aligned around germinal centers. The germinal center is indicated by GC and dotted circles with a scale bar of 50 μm. Immunohistochemical analyses by cyclin D1, with a scale bar of 50 μm.

The tumor macroscopically showed a submucosal polypoid appearance (Figure 2A). Resected tissue was fixed in 20% formalin, embedded in paraffin, and routinely stained with hematoxylin and eosin, followed by immunohistochemical analyses with monoclonal antibodies, as summarized in Table 1 (supplement). The tumor was composed of multiple, vague nodules (Figure 2A,2B) that contained abortive CD10+Ki67+ germinal centers surrounded by a regular proliferation of CD3-CD20+CD79a+bcl2+ small lymphoid cells without immunoblastic large cells (Figure 2C). These areas corresponded to the mantle zone, with no definite formation of the marginal zone observed. No lymphoepithelial lesions (intraepithelial infiltration of neoplastic B-cells with architectural distortion of epithelial structures) were observed, but it showed a typical mantle cell pattern, which was further supported by a sparse distribution of CD21+ follicular dendritic cells (FDCs) next to a dense distribution of FDC in the germinal centers (Figure 3) and negativity for MUM1 protein (approximately 1%, data not shown) [3]. We then examined the chromosomal rearrangement involving IgH and Bcl1 by fluorescence *in situ* hybridization. In brief, DNA was hybridized with Vysis^®^ LSI^®^ IGH/CCND1 Rearrangement probes (ABBOTT, Wiesbaden, Germany), as described previously [4]. The IgH-BclI chimera was observed in only 1% of examined cells (2.8%, with 0.8% standard deviation, in 10 healthy volunteers). Proliferating cells stained for cyclin D1, D2, or D3 only sporadically (1-2%) without expressing IgD or IgM (data not shown). We noted infiltration by CD5+ T cells, but the majority of proliferating cells were negative for CD5 (Figure 3). This pattern was also similar to the mantle zone of the normal lymphoid tissue (Figure 3).

**Table 1.** List of antibodies used in this study

Antibodies for	Spieces	Sources	Clones	Final dilution	Heat antigen retreival
CD3	mouse Mab	Dako	F7.2.38	1:40, 6 mg/ml	T/E, 95C, 60 min
CD5	mouse Mab	Novocastra	4C7	1:100	T/E, 95C, 60 min
CD10	mouse Mab	Novocastra	56C6	1:100	T/E, 95C, 60 min
CD20	mouse Mab	Dako	L26	1:400, 1 mg/ml	none
CD21	mouse Mab	Dako	1F8	1:50, 7 mg/ml	Dako S1700, 95C, 60min
CD79a	mouse Mab	Nichirei	JCB117	1:150	T/E, 95C, 60 min
bcl-2	mouse Mab	Dako	124	1:100, 2.6 mg/ml	T/E, 95C, 60 min
MUM1 protein	mouse Mab	Dako	MUM1p	1:100, 7.5 mg/ml	T/E, 95C, 60 min
cyclin D1	rabbit Mab	Nichirei	SP4	1:25	T/E, 95C, 60 min
cyclin D2	rabbit Pab	Santa Cruz	(sc-593)	1:100, 2ug/ml	T/E, 95C, 60 min
cyclin D3	mouse Mab	Progen	DCS-22	undiluted	T/E, 95C, 60 min
IgD	rabbit Pab	Dako	A0093	1:1500	RT, 30 min

Mab, monoclonal antibody. Pab, polyclonal antibody. RT, room temperature T/E, 10mM Tris HCl buffer/1mM EDTA, pH9.0 Dako, Glustrup, Denmark.

Novocastra, Benton Lane, UK. Nichirei, Tokyo, Japan. Santa Cruz Biotechnology, Santa Cruz, CA. Progen Biotechnik, Heidelberg, Germany.

The patient experienced a good post-operative course, and was enrolled in the Department of Hematology. Lymphoma metastasis was evaluated by a whole body CT scan, PET-CT, bone marrow examination, Ga-scintigram, and total colonoscopy, but no other sites were involved. The diagnosis was stage IAE, and rituximab was given as adjuvant chemotherapy. This patient has been disease free for more than 28 months after surgery.

## Discussion

Primary esophageal lymphoma is rare, with no MCL reported except a case in cardiac-type glandular tissue replacement of the lower esophagus [1,2,5-7]. This case, reported by Reyes, contains the typical molecular features of MCL, CD5 staining, cyclin D1 staining, and chromosomal translocation [t(11;14)(q13;q32)] [2]. However, this case arose in cardiac-type glandular tissue replacing the lower oesophagus, not in squamous epithelial tissue. Furthermore, MCL could have originated in the herniated subcardial region of the stomach, because the diagnosis was obtained from biopsies and the presence of a proper esophageal gland was not confirmed.

MALT lymphoma can arise in the esophagus. Hosaka and colleagues showed that three cases previously reported as a B-cell type non-Hodgkin's lymphoma might be MALT lymphomas [1]. The differential diagnosis of malignant lymphoma could improve with endoscopic mucosal resection, which obtains a greater amount of tissue than conventional biopsies. Our case showed recurring proliferation of small lymphocytes, but no large cells, surrounding abortive germinal centers. This indicates proliferation of the mantle zone but not the marginal zone. For differential diagnosis, we first need to consider extranodal marginal zone lymphoma (MALT lymphoma). However, the marginal zone typically seen in MALT lymphoma was not present here [1], nor was there a lymphoepithelial lesion. Therefore, we excluded MALT lymphoma. We also need to consider Castleman disease, which includes a hyaline vascular type and a plasma cell type [8,9]. The germinal center here lacked the characteristic vasculature with a hyalinized wall, and plasma cell infiltration was not observed in the whole areas. These pathological findings prompted us to exclude Castleman's disease.

Although this case resembled MCL, it lacked the immunostaining and molecular features typical for MCL, including weak expression of cyclins D1, D2, D3, CD5, IgD, or IgM. According to the WHO classification 4^th^ edition, cyclin-D1-negative MCL should overexpress cyclin D2 or D3, as confirmed by gene expression profiles [10]. In addition, MCL prognosis is not favourable, with the vast majority of MCL patients not curable long-term [11]. The current case has been disease free for more than 28 months, suggesting that this case clinically does not fit typical MCL.

## Conclusion

Taken together, the current case has similar histological features to MCL, but does not fit the MCL diagnosis. The present case may be “hyperplasia of the mantle zone without neoplastic transformation,” but this conclusion would require careful follow-up. We also need further clinicopathological data regarding B-cell lymphomas of the esophagus.

## List of abbreviations

CT: Computed tomography
GI: Gastro-intestinal
MALT: Mucosa-associated lymphoid tissue
MCL: Mantle cell lymphoma
PET-CT: Computed tomography
